# A rare case of musculocontractural Ehler-Danlos syndrome with cornea plana and juvenile glaucoma

**DOI:** 10.1016/j.ajoc.2026.102556

**Published:** 2026-03-10

**Authors:** Madhuri Manapakkam, P.V. Manjusha, Deepa Ramamoorthy

**Affiliations:** Department of Glaucoma Services, Aravind Eye Hospital, Chennai, India

## 1 Case report

**Fig. 1:**
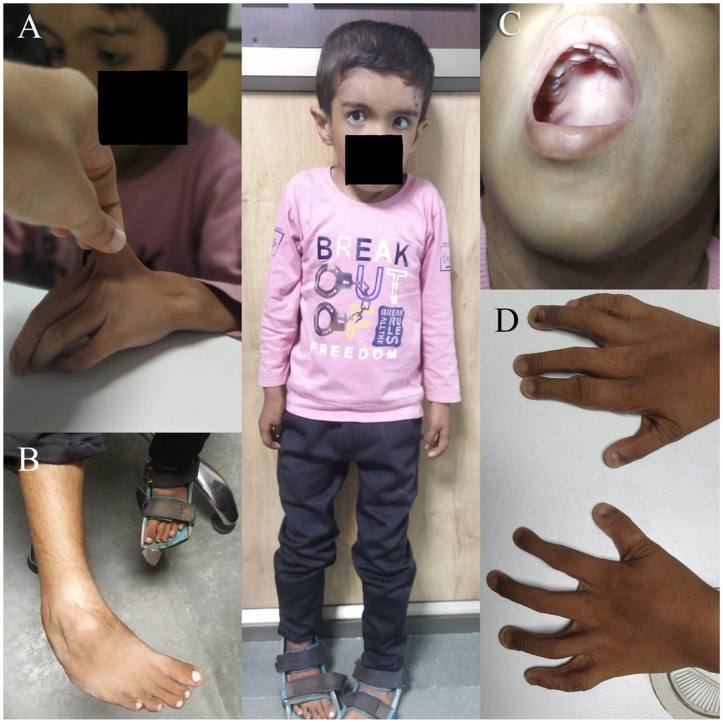
Systemic features of mc-EDS A) Hyperextensible skin B) Broad surgical scar post CTEV correction C) High arched palate D) Long and slender fingers with joint laxity.

**Fig. 2:**
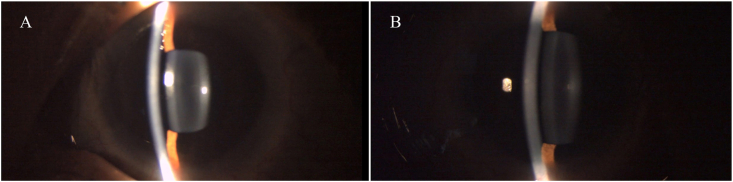
Slit lamp picture showing cornea plana in both eyes.

A 6-year-old male child with Musculocontractural Ehler–Danlos syndrome (mcEDS) was referred to our outpatient department with a complaint of defective vision in both eyes for a period of 6 months. On examination, his best corrected visual acuity was 6/60 in both eyes and was found to be a high myope with a −12-dioptre sphere and −6 dioptre cylinder at 90° in the right eye and a −11.5-dioptre sphere and −6 dioptre cylinder at 80° in the left eye as confirmed by retinoscopy. The child presented with a flat nose, epicanthal folds, and pseudoesotropia in the left eye (Fig. 1). Intraocular pressure (IOP) in the right and left eyes was 34 and 33 mmHg, respectively. Anterior segment examination revealed cornea plana in both eyes (Fig. 2) with keratometry K1 and K2 readings being 34.03 D, 38.78 D in the right eye (OD) and 34.41 D, 38.58 D in the left eye (OS). Central corneal thickness (CCT) measured 492 μm in OD and 488 μm in OS. Axial length measured was 29.40 mm in OD and 29.13 mm in OS, while the horizontal corneal diameter measured 10.8 mm in OD and 10.6 mm in OS.

**Fig. 3:**
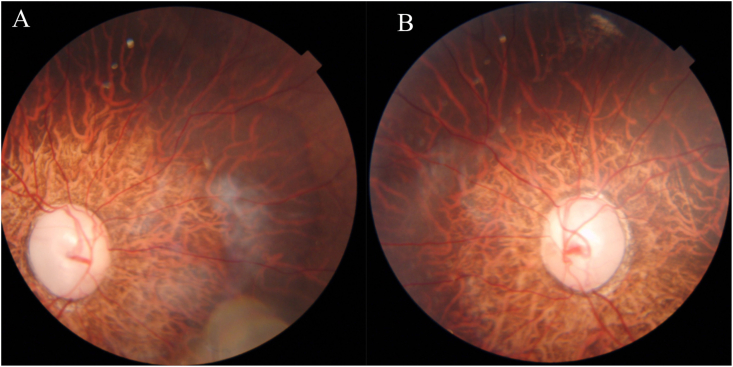
Fundus photo showing large myopic disc with a cup-disc ratio of 0.9:1 and marked neuroretinal rim thinning, along with peripapillary atrophy and a tessellated fundus.

Anterior segment OCT showed open angles in both eyes. Fundus examination of both eyes revealed a large myopic optic discs with a cup-disc ratio of 0.9:1 and marked neuroretinal rim thinning, along with peripapillary atrophy and a tessellated fundus consistent with axial myopia (Fig. 3). There is no family history of ocular conditions, specifically cornea plana, congenital glaucoma, or juvenile glaucoma. Additionally, there was no known family history of Ehlers–Danlos syndrome. The child's IOP was initially controlled with multiple topical antiglaucoma medications; however, surgical intervention was required after 3 months due to inadequate IOP control. Trabeculectomy was performed in both eyes without adjunctive Mitomycin C to minimize the risk of hypotony and bleb-related complications in view of the scleral fragility associated with Ehlers–Danlos syndrome. Postoperatively, the best-corrected visual acuity remained 6/60 in both eyes, while intraocular pressure was well controlled at 9 mmHg in the right eye and 10 mmHg in the left eye. Genetic testing revealed a homozygous base pair deletion in exon 1 of the ***CHST14*** gene. This variant is classified as likely pathogenic, supporting a diagnosis of Ehlers–Danlos syndrome, musculocontractural type 1 (OMIM#601776).

## 2 Discussion

Musculocontractural Ehler–Danlos syndrome is a rare subtype of Ehler–Danlos syndrome that has been reported in fewer than 100 patients worldwide.^1^ It is an autosomal recessive connective tissue disorder caused by biallelic mutations in CHST14 or in DSE, which encodes for the enzyme carbohydrate sulfotransferase 14 and dermatan sulfate epimerase, respectively, which are involved in dermatan sulfate (DS) biosynthesis. The relative absence of dermatan sulfate leads to an excess of chondroitin sulfate, which ultimately disrupts the normal structure of collagen throughout the body and results in abnormal regulation of collagen fibril assembly.^2^ In genetic testing of our patient a homozygous c.559del (p.Asp187ThrfsTer55) base pair deletion in exon 1 of the CHST14 gene (chr15:g.40471772del; Depth: 80x) that results in a frameshift and premature truncation of the protein 55 amino acids downstream to codon 187 (p.Asp187ThrfsTer55; ENST00000306243.7) was identified and has not been reported in the 1000 genomes, gnomAD (v3.1), gnomAD (v2.1) and topmed databases. The frameshift variant results in premature truncation at 242 amino acids (187 + 55). There is a loss of more than 10% of the protein. Hence, as per ACMG (American College of Medical Genetics and Genomics) 2015 criteria, the gene detected is a novel variant. Based on the above evidence, this CHST14 variation detected is classified as a likely pathogenic variant. Genes associated with congenital and juvenile glaucoma were analysed in the NGS (Next-generation sequencing) data, and no significant variants were detected. These findings support a diagnosis of Ehlers–Danlos syndrome, musculocontractural type 1 (OMIM#601776). The identified mutation correlates with the child's phenotypic features, further strengthening the association between the ocular findings and mcEDS.

Musculocontractural Ehler–Danlos syndrome is characterized by congenital multiple contractures (adducted thumbs, talipes equinovarus), skin fragility hyperextensibility, joint laxity, acrogeria and characteristic craniofacial features including large fontanelle, hypertelorism, short and down slanting palpebral fissures, blue sclerae, short nose with hypoplastic columella, low set and rotated ears, high arched palate, long philtrum, thin upper lip vermilion, small mouth and micro retrognathia.^3^ Collagen is a critical structural protein that supports the integrity of various ocular tissues. In mcEDS, the abnormal collagen fibril assembly compromises the biomechanical stability of the eye. In the cornea, such abnormalities may result in altered curvature and thinning, contributing to conditions like cornea plana. Corneal involvement across EDS subtypes is heterogeneous and may include diverse manifestations like keratoconus, microcornea, sclerocornea, marked corneal flattening and features consistent with cornea plana. This reflects the phenotypic variability of corneal involvement across EDS subtypes and emphasises the importance of careful corneal assessment in these patients. Similarly, defective collagen in the trabecular meshwork may impair aqueous outflow, leading to elevated intraocular pressure and the development of glaucoma. The reduced visual acuity in this patient may be partly attributable to refractive amblyopia secondary to high uncorrected refractive error during the critical period of visual development, in addition to glaucomatous optic neuropathy. This underscores the importance of prompt ophthalmic referral for early cycloplegic refraction, and amblyopia management in such paediatric, syndromic patients for better long term visual outcomes. This case highlights that patients with mcEDS can present with complex ocular associations, including the coexistence of high myopia, cornea plana, and juvenile-onset glaucoma, necessitating comprehensive ophthalmic management. This unique combination expands the current understanding of ocular manifestations in mcEDS and underscores the importance of early ophthalmic evaluation in these patients.

## 3 Conclusion

This case underscores the critical need to raise awareness among paediatricians and general physicians about the importance of routine ophthalmic evaluations in children with mcEDS, enabling early detection and management of potentially sight-threatening ocular manifestations. Greater awareness among primary care providers could be the key to preserving vision and preventing irreversible vision loss.

A better understanding of mcEDS is essential for effective management and may significantly improve the patient's quality of life. Proactive, multidisciplinary care can help reduce the burden of this complex condition and alleviate the psychological stress experienced by both patients and their families.

## CRediT authorship contribution statement

**Madhuri Manapakkam:** Conceptualization, Data curation, Investigation, Formal analysis, Review and editing, Supervision. **P.V. Manjusha:** Writing – original draft, Analysis, Review & editing. **Deepa Ramamoorthy:** Investigation, Analysis, Writing – review & editing.

## Patient consent

Written consent to publish this case report has been obtained from the patient's parent.

## Authorship

All authors attest that they meet the current ICMJE criteria for Authorship.

## Funding

There was no funding or grant support for this report.

## Declaration of competing interest

The authors declare that they have no known competing financial interests or personal relationships that could have appeared to influence the work reported in this paper.
